# Identification of Novel Molecular Therapeutic Targets and Their Potential Prognostic Biomarkers Among Kinesin Superfamily of Proteins in Pancreatic Ductal Adenocarcinoma

**DOI:** 10.3389/fonc.2021.708900

**Published:** 2021-09-07

**Authors:** Yang Yang, Lanyang Gao, Ning-Na Weng, Jun-Jun Li, Jin Lu Liu, Ying Zhou, Rong Liao, Qun-Li Xiong, Yong-Feng Xu, Armando Varela-Ramirez, Qing Zhu

**Affiliations:** ^1^Department of Abdominal Oncology, West China Hospital of Sichuan University, Chengdu, China; ^2^Sichuan Provincial Center for Gynaecology and Breast Disease, The Affiliated Hospital of Southwest Medical University, Southwest Medical University, Luzhou, China; ^3^Department of Biological Sciences, The Border Biomedical Research Center (BBRC), The University of Texas at El Paso, El Paso, TX, United States

**Keywords:** bioinformatics tools, kinesin superfamily of proteins, pancreatic cancer, TCGA, overall survival

## Abstract

**Background:**

Kinesin superfamily of proteins (KIFs) has been broadly reported to play an indispensable role in the biological process. Recently, emerging evidence reveals its oncogenic role in various cancers. However, the prognostic, oncological, and immunological values of KIFs have not been comprehensively explored in pancreatic ductal adenocarcinoma (PDAC) patients. We aimed to illustrate the relationship between KIFs and pancreatic ductal adenocarcinoma by using bioinformatical analysis.

**Methods:**

We use GEPIA, Oncomine datasets, cBioPortal, LOGpc, TIMER, and STRING bioinformatics tools and web servers to investigate the aberrant expression, prognostic values, and oncogenic role of KIFs. The two-gene prognostic model and the correlation between KIFs and KRAS and TP53 mutation were performed using an R-based computational framework.

**Results:**

Our results demonstrated that KIFC1/2C/4A/11/14/15/18A/18B/20B/23 (we name it prognosis-related KIFs) were upregulated and associated with unfavorable clinical outcome in pancreatic cancer patients. KIF21B overexpression is associated with better clinical outcome. The KIFC1/2C/4A/11/14/15/18A/18B/20B/23 profiles were significantly increased compared to grade 1 and grade 2/3. Besides, KIFC1/2C/4A/11/14/15/18A/18B/20B/23 was significantly associated with the mutation status of KRAS and TP53.Notably, most prognosis-related KIFs have strong correlations with tumor growth and myeloid-derived suppressor cells infiltration (MDSCs). A prognostic signature based on KIF20B and KIF21B showed a reliable predictive performance. Receiver operating characteristic (ROC) curve was employed to assess the predictive power of two-gene signature. Consequently, the gene set enrichment analysis (GSEA) showed that KIF20B and KIF21B’s overexpression was associated with the immunological and oncogenic pathway activation in pancreatic cancer. Finally, real-time quantitative PCR (RT-qPCR) was utilized to investigate the expression pattern of KIF20B and KIF21B in pancreatic cancer cell lines and normal pancreatic cell.

**Conclusions:**

Knowledge of the expression level of the KIFs may provide novel therapeutic molecular targets and potential prognostic biomarkers to pancreatic cancer patients.

## Introduction

Pancreatic adenocarcinoma (PDAC) accounts for 85% of all pancreatic cancer, and it is one of the most lethal digestive duct malignancies with an increasing incidence in recent years (1). It is estimated to be the second deadliest cancer in the United States by 2025 (2). Surgical resection offers only possibility for a cure at present. However, most of the patients have lost the opportunity for surgery when they were diagnosed due to the inability of early detecting of tumors, high malignancy, and high risk of metastasis. At present, the principal treatment option for advanced patients is gemcitabine-based chemotherapy, but it only has a limited effect on long-term survival. Although immunotherapy has shown unprecedented response rates in some recalcitrant cancers, the clinic benefit of immunotherapy in PDAC such as PD-1/L1 and CTLA-4 is still dismal (3). Hence, uncovering more potential functions of known molecules will provide new therapeutic targets and indicators of prognosis.

Kinesin superfamily of proteins (KIFs) are known as molecular motors with microtubule (MT) class-binding protein superfamily, which plays a crucial role in biological processes, including cell division (4), intracellular transport (5), microtubule stabilization (6), and microtubule depolymerizers (6). KIFs include 14 families (kinesin-1 to kinesin-14) and 45 members, which exert various biological functions in our body, especially in the brain (7–9). For instance, the kinesin 4 family motor KIF4A is required for activity-dependent neuronal survival (10). KIF20A was reported to play an indispensable role in cell division and Golgi-derived vesicle transportation (11). In addition, KIF21A and KIF26A are responsible for microtubule stabilizers (12, 13), while KIF2A and KIF19A are implicated in microtubule depolymerizers (14). In the past few years, numerous studies have indicated that kinesin’s aberrant expression is involved in development and progress many kinds of cancers (15–19). As a member of KIFs, KIF18A can independently predict unfavorable prognosis in lung adenocarcinoma; overexpression of KIF18A can promote cell proliferation and inhibit apoptosis (20). Moreover, KIF21B is upregulated in NSCLC and acts as an oncogene, which promotes the growth and metastasis of NSCLC (16). Besides, aberrant expressions of KIF1B (15), KIF20A (11), KIFC (21), and KIF2C (22) proteins were detected in gastric cancer, which exhibits diverse mechanisms in carcinogenesis and cancer progression.

In our study, we carried out an integrated analysis to better understand the oncogenic role of KIFs in the development of pancreatic cancer. A guided flowchart of the strategy and methodology is shown in **Supplementary Figure 3**. We first screened KIFs genes, all of which have the characteristic of high expression in PDAC and are associated with prognosis as our candidates based on both GEPIA and Oncomine databases. Finally, 12 prognosis-related KIFs were included for further study. Genomic alterations in prognosis-related genes were analyzed to determine the mutation status of prognosis-related KIF and overall patient survival. We performed a series of correlation analyses to explore potential roles of 12 prognosis-related KIFs. We then continued to examine the correlations between mutation status of KRAS and TP53 and the parameters, including tumor grade, immune infiltration, and cell growth. To further explore the prognosis value of prognosis-related KIFs, we utilized statistical methods to construct a two-gene prognostic model. Protein–protein interaction (PPI) of two gene products, and some additional related gene products, was also performed *via* GSEA computational method to investigate its precise mechanism involved in tumor progression.

## Materials and Methods

### Identification of Aberrantly Expressed Kinesin Superfamily Genes in PDAC

GEPIA (http://gepia.cancer-pku.cn/index.html) is an online tool used for integrating analysis of gene expression data from TCGA (23). In our study, GEPIA was employed to analyze the aberrant gene expression of 45 kinesin superfamily members in PDAC. All the results were presented as box plots by comparing TCGA pancreatic cancer and GTEx data. The significantly up- or downregulated genes are marked with “*”, which means *p* < 0.05.

Oncomine datasets (www.oncomine.org) is a publicly accessible online cancer microarray database used to validate the expression patterns of the prognosis-related KIFs (24, 25). Student’s *t*-test was utilized to compare the transcription levels of KIFs in pancreatic cancer specimens with those in normal controls. The cutoffs of *p*-value and fold-change were defined as 0.05 and 1.5, respectively.

### Survival Analysis

GEPIA is a multifunctional online tool that can perform OS or disease-free survival (DFS, also called relapse-free survival and RFS) analysis based on gene expression (23). Here, the GEPIA overall survival analysis was performed to evaluate kinesin superfamily genes’ prognostic values. The Cox proportional hazard ratio and 95% confidence interval information were also included in the survival plot.

LOGpc (http://bioinfo.henu.edu.cn/DatabaseList.jsp) is an online server that encompasses 209 expression datasets for survival analysis. It provides 27 types of malignant tumors for 31,310 cancer patients. Its patient samples were mainly derived from TCGA and GEO cohorts (26). The LOGpc datasets were then performed by combined overall survival analysis of prognosis-related kinesin superfamily genes to reaffirm the genes’ predictive values in PDAC. When the *p*-value of the combined overall survival is <0.05, it is regarded as a significant gene.

### Tumor Grade Correlation With Prognosis-Related KIFs

UALCAN (http://ualcan.path.uab.edu) is an online platform aimed to perform silicon validation of potential genes of interest and clinical–pathological parameters, such as tumor grade (27). Box–whisker plot was then employed to virtualize the correlation between the tumor grade and kinesin superfamily expression patterns. The statistical significance of the two samples was accomplished using the Student’s *t*-test, and a *p*-value like to or <0.05 was deemed significant.

### Kinesin Superfamily Genomic Alterations in PDAC

cBioPortal (https://www.cbioportal.org/) is an online platform applying to visualize analysis and download large-scale cancer genomics datasets (28). In our study, cBioPortal was utilized to analyze genetic alterations containing alterations rate and the categories of genetic alterations of each prognosis-related KIFs in PDAC.

### KRAS, TP53 mutation, and Prognosis-Related KIFs

Data of mutations about TP53 and KRAS were downloaded from cBioPortal database (http://www.cbioportal.org/) (29). We then combined these mutation data with the gene expression data of prognosis-related data downloaded from UCSC Xena (https://xena.ucsc.edu/) to do analysis. Wilcoxon rank-sum test was employed to clarify the relation between KRAS, TP53 mutation, and kinesin superfamily members. Violin plot was archived using the R package of “ggpubr” in an R environment (R version: 4.02). A *p*<0.05 is regarded as statistical significance.

### Prognosis-Related KIFs and Tumor Proliferation

The well-known proliferation marker ki67 was employed to reflect tumor proliferation in PDAC samples downloaded from UCSC Xena. We then assessed the association between individual prognosis-related KIFs and proliferation ability by Spearman’s correlation, and |Rs| > 0.2, and FDR < 0.05 was considered as statistical significance (30). CCLE database (https://portals.broadinstitute.org/ccle/about) is applied to reaffirm the proliferation promotion role of prognosis-related KIFs in various pancreatic cell lines (31).

### Immune Infiltration and Prognosis-Related KIFs

TIMER (https://cistrome.shinyapps.io/timer/) is a web resource for systematical evaluations of the clinical impact of six immune cell types: B cell, CD4 T cell, CD8 T cell, neutrophil, macrophage, and dendritic cell in diverse cancer types (32). We then utilized the correlation module of TIMER to illustrate the relationships between prognosis-related kinesin superfamily and immune cell infiltration. A purity-corrected partial Spearman’s rho value and statistical significance were presented in expression scatter plots. We performed a similar analysis between the CD274 (also known as PD-L1) and kinesin superfamily. TIMER2 (http://timer.cistrome.org/), the latest version of TIMER, was applied to clarify the relationship between immune cell infiltration and myeloid-derived suppressor cells (33).

TISIDB database (http://cis.Hku.hk/TISIDB/) is a web portal for comprehensive investigation of tumor–immune interactions, which integrate multiple heterogeneous data types (34). We analyze the correlation between prognosis-related KIFs expression and tumor-infiltrating lymphocytes in this platform.

### PPI Analysis and the Similar Genes of KIF20B and KIF21B in PDAC

Cytoscape is an open-source software platform for visualizing complex networks and integrating these with any type of attribute data (35). We first retrieved 40 similar genes of KIF20B and KIF21B through expression analysis of GEPIA (http://gepia.cancer-pku.cn/detail.php?gene=ATM). Those repeated genes were deleted, and we further analyzed the protein–protein interaction of KIF20B and KIF21B and their similar genes in STRING, an online tool for performing protein–protein interaction networks analysis. Finally, the interaction network was then imported to Cytoscape to virtualize protein–protein interaction.

### The Construction of Prognostic Model

The TCGA expression data and clinic data were downloaded from UCSC Xena, datasets that collect public databases, including TCGA, TARGET, GTEx, ICGC, and CCLE (36). To ensure the quality of our analysis, the annotations from PanCanAtlas Publications were employed to filter the unqualified samples. Eight neuroendocrine samples, 11 samples <1% neoplastic cellularity, 2 IPMN, 1 acinar cell carcinoma sample, 1 systemic treatment given to the prior/other malignancy sample, and 1 sample arise from ampulla were excluded from this study. The patients whose overall survival time <1 month were excluded from our further investigation. Then, univariate cox regression analysis was performed to identify several genes as candidate markers correlated with PDAC prognosis. LASSO regression using 10-fold cross-validation analysis were employed to refine these genes. The refined genes were extracted to perform multivariate regression analysis. The risk score was calculated by the following formula: (1.067*KIF20B exp.) + (−0.765*KIF21B exp.).

TCGA-PAAD was divided into low- and high-risk groups according to the median risk score. The survival differences between two groups were compared and visualized with the survival status plot, risk heatmap, and K–M plotter. AUC of the 95% confidence interval was calculated based on the ROC curve. The accuracy and specificity of the two-gene model were evaluated. Diagnosis of age, gender, histological grade, stage, and risk score were all included for univariate and multivariate Cox regression analysis, which determined that risk score based on KIF20B and KIF21B was the independent prognostic factor for PDAC.

### Gene Set Enrichment Analysis

To further explore underlying mechanisms of the two prognosis-related genes, GSEA (https://www.gsea-msigdb.org/gsea/index.jsp) was performed in this study (37). We then divided patient samples into two groups with high and low expression levels, respectively, according to median expression value of prognosis-related genes. The Molecular Signatures Database (MSigDB) used in this research are as follows: C2 (c2.cp.kegg.v7.2.symbols.gmt), C5 (c5.all.v6.2.symbols.gmt), C6 (c6.all.v6.2.symbols.gmt), and C7 (c7.all.v7.3.symbols.gmt). The nominal *p* < 0.05, FDR < 0.25, and enrichment score (NES) > 1.5 were defined as the significantly enriched gene sets.

### Cell Culture, RNA Extraction, and qRT-PCR

ASPC-1, BxPC-3, Capan-1, Capan-2, CFPAC-1, MIA PACA-2, PANC-1, and hTERT-HPNE were obtained from the Cell Bank of the Shanghai Institute of Cells, Chinese Academy of Science (Shanghai, China). RNA was isolated from cell lines using cell total RNA extraction kit (Cat. No. RE-03113F, ORGENE, China) according to the manufacturer’s instructions. The complementary DNA (cDNA) was synthesized using the PrimeScript™ RT Master Mix (Cat. No. RR036A, TaKaRa, Japan). Real-time quantitative PCR (RT-qPCR) was performed using NovoStart^®^ SYBR qPCR SuperMix Plus (Cat. No. E096-01B, Novoprotein, China). The specific primers used were as follows: glyceraldehyde 3-phosphate dehydrogenase (GAPDH) forward, GTCTCCTCTGACTTCAACAGCG; reverse, ACCACCCTGTTGCTGTAGCCAA; KIF20B forward, CCGGGAAAGTAAACTGACTCAC; reverse, TTCTAGCTCCTCAACCAAATCCT; KIF21B forward, AGAACAGCGAGGAGACGGATGA; reverse, TCTGAGTCCACCAGGCTCTCTT.

The amplification reaction included the following steps: 95°C for 1 min, followed by 39 cycles of 95°C for 20 s and 60°C for 1 min. GAPDH was used as an internal control for mRNA, and the relative expression level of mRNAs was calculated by the relative quantification (2^−ΔΔCt^) method.

## Results

### Kinesin Superfamily and Its Expression Patterns in Pancreatic Cancer

The expression patterns of KIFs were illustrated through public available datasets. We also used GEPIA to explore the kinesin superfamily gene expression in pancreatic adenocarcinoma samples from The Cancer Genome Atlas (TCGA) compared to Genome Tissue Expression (GTEX) (normal). Box plots were generated for 45 genes encoding kinesin superfamily (KIF11, KIF12, KIF13A, KIF13B, KIF14, KIF15, KIF16B, KIF17, KIF18A, KIF18B, KIF19, KIF1A, KIF1B, KIF1C, KIF20A, KIF20B, KIF21A, KIF21B, KIF22, KIF23, KIF24, KIF25, KIF25-AS1, KIF26A, KIF26B, KIF27, KIF28P, KIF2A, KIF2B, KIF2C, KIF3A, KIF3B, KIF3C, KIF4A, KIF4B, KIF4CP, KIF5A, KIF5B, KIF5C, KIF6, KIF7, KIF9, KIF9-AS1, KIFAP3, KIFC1, KIFC2, KIFC3) (**Supplementary Figure 1**).

**Figure 1:**
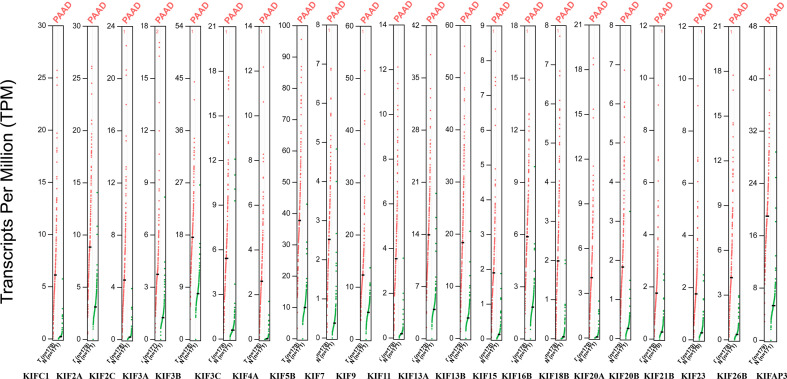
The expression of KIFs in PDAC (GEPIA). Dot plots profiling gene expression between cancer samples (red dots) and paired normal samples (green dots), with each dot representing a distinct tumor or non-cancerous samples. Each column represents a gene. The genes that are differential expression in cancer tissues compared to normal tissues were presented above (*p* < 0.05). The TPM value is used to display the relative expression of KIFs.

We observed that most of the 45 genes encoding kinesin superfamily have a statistically upregulation in tumor samples (KIFC1, 2A, 2C, 3A, 3B, 3C, 4A, 5B, 7, 9, 11, 13A, 13B, 15, 16B, 18B, 20A, 20B, 21B, 22, 23, 26B, and AP3) (*p* < 0.05; **Figure 1**). Only KIF1A has exhibited a statistically significant decrease in tumor tissues compared to normal tissues. All the rest of genes do not harbor any significant expression variations. Furthermore, the Oncomine datasets were utilized to reaffirm the aberrant expression of KIFs. We noted a similar expression patterns with a significant increase in KIFC1, 2A, 2C, 3A, 3C, 4A, 4B, 5B, 11, 13A, 13B, 15, 16B, 18B, 20A, 20B, 21B, 22, 23, 26B, and AP3 (**Table 1**). There is also a decrease in the expression of genes encoding KIF1A protein (**Table 1**). In contrast, both of KIF3B and KIF7 have statistical significance according to Oncomine dataset, with a *p* value of 0.065 and 0.068, respectively (**Table 1**).

**Table 1:** The significant fold changes of KIFs expression in transcription level between different types of PDAC and normal pancreatic tissues (Oncomine).

**Genes**	**Types of PDAC vs. normal**	**Fold change**	***t*-test**	***p*-value**	**Ref**
KIFC1	Pancreatic Carcinoma	1.534	3.896	2.05E−4	Pei Pancreas (38)
KIF2A	Pancreatic Carcinoma	1.531	3.244	0.002	Pei Pancreas (38)
	Pancreatic Ductal Adenocarcinoma	1.600	4.547	1.69E−5	Badea Pancreas (39)
KIF2C	Pancreatic Carcinoma	2.998	6.606	2.68E−8	Pei Pancreas (38)
KIF3A	Pancreatic Ductal Adenocarcinoma	1.892	8.096	7.29E−12	Badea Pancreas (39)
KIF3B	Pancreatic Ductal Adenocarcinoma	1.443	1.677	0.065	Iacobuzio-Donahue Pancreas (40)
KIF3C	Pancreatic Carcinoma	1.626	2.775	0.005	
	Pancreatic Ductal Adenocarcinoma	1.407	6.619	3.64E−9	Pei Pancreas (38)
KIF4A	Pancreatic Carcinoma	2.964	7.160	1.12E−8	Badea Pancreas (39)
KIF5B	Pancreatic Carcinoma	1.543	3.219	0.002	Pei Pancreas (38)
KIF7	Pancreatic Carcinoma	1.084	1.523	0.068	Pei Pancreas (38)
KIF9	Pancreatic Carcinoma	1.210	2.193	0.018	Pei Pancreas (38)
KIF11	Pancreatic Carcinoma	3.635	6.599	1.63E−7	Pei Pancreas (38)
KIF13A	Pancreatic Carcinoma	1.481	3.513	3.76E−4	Pei Pancreas (38)
KIF13B	Pancreatic Carcinoma	2.169	4.392	8.29E−5	Badea Pancreas (39)
KIF15	Pancreatic Ductal Adenocarcinoma	1.804	3.357	0.002	Pei Pancreas (38)
	Pancreatic Carcinoma	1.718	4.361	3.40E−5	Grutzmann Pancreas (41)
KIF16B	Pancreatic Carcinoma	1.663	2.188	0.020	Pei Pancreas (38)
KIF18B	Pancreatic Carcinoma	2.239	5.742	2.75E−7	Pei Pancreas (38)
KIF20A	Pancreatic Ductal Adenocarcinoma	2.656	8.693	8.64E−7	Pei Pancreas (38)
KIF20B	Pancreatic Carcinoma	2.561	5.212	2.40E−6	Buchholz Pancreas (42)
KIF21B	Pancreatic Carcinoma	1.198	2.786	0.004	Pei Pancreas (38)
KIF22	Pancreatic Adenocarcinoma	1.792	2.565	0.018	Pei Pancreas (38)
KIF23	Pancreatic Carcinoma	1.645	4.466	4.76E−5	Iacobuzio-Donahue Pancreas (40)
	Pancreatic Carcinoma	2.104	6.324	4.07E−8	Pei Pancreas (38)
					Pei Pancreas (38)
	Pancreatic Ductal Adenocarcinoma	2.845	2.031	0.029	Grutzmann Pancreas (41)
KIF26B	Pancreatic Carcinoma	1.337	3.288	0.001	Pei Pancreas (38)
KIFAP3	Pancreatic Ductal Adenocarcinoma	1.473	6.213	2.23E−8	Badea Pancreas (39)

### KIFs and Patient Survival

**Figure 2:**
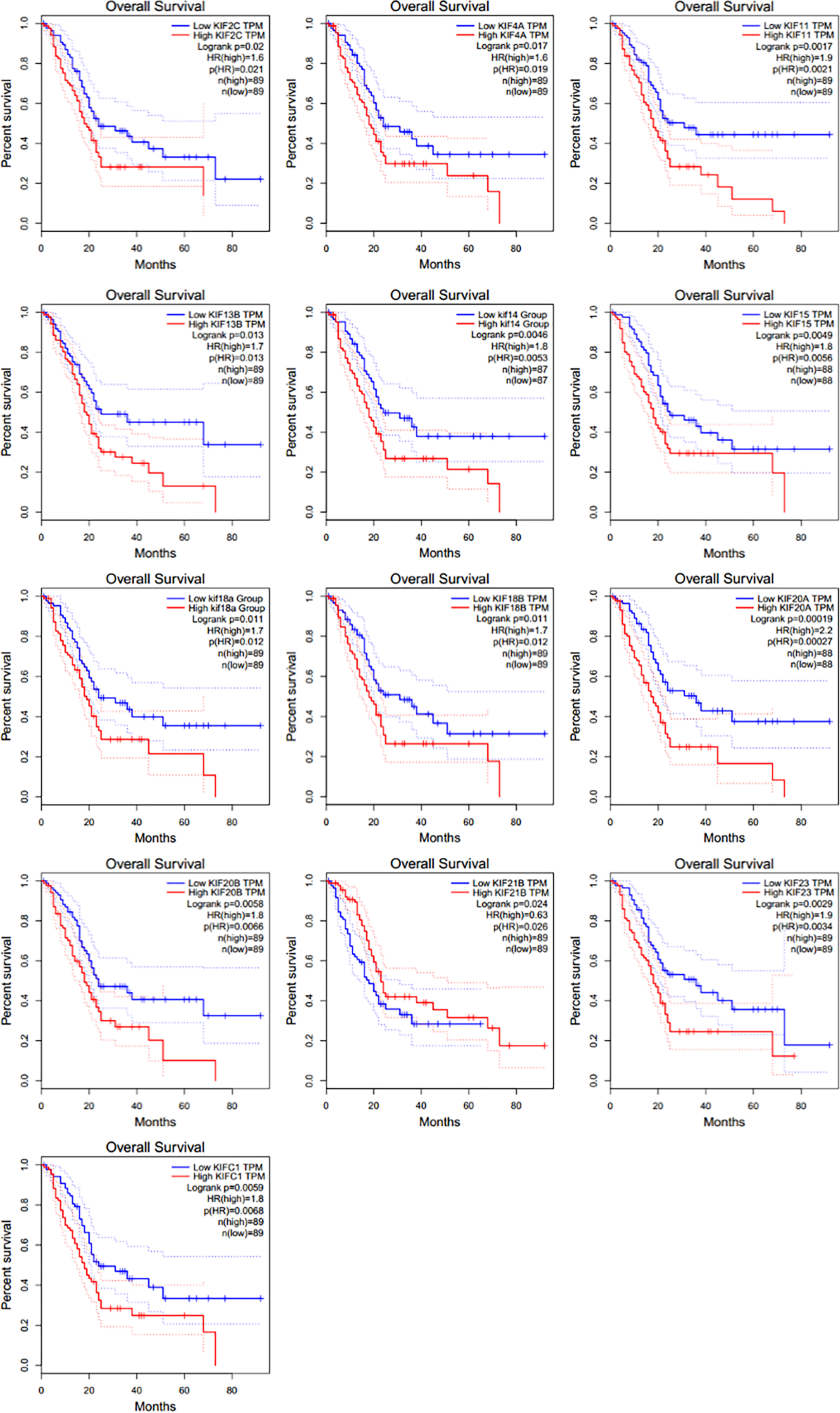
The prognostic value of KIFs in PDAC patients in the OS curve (GEPIA). Overall survival analyses of 13 prognostic genes (KIF2C, KIF4A, KIF11, KIF13B, KIF14, KIF15, KIF18A, KIF18B, KIF20A, KIF20B, KIF21B, KIF23, and KIFC1) at pancreatic cancer based on the GEPIA database.

We then investigated the association between expression of KIFs genes and patient survival using GEPIA tool. It was noticed that poorer patient survival is significantly associated with the high expression of KIFC1 (HR = 1.8), KIF2C [hazard ratio (HR) = 1.6], KIF4A (HR = 1.6), KIF11 (HR = 1.9), KIF13B (HR = 1.7), KIF14 (HR = 1.8), KIF15 (HR = 1.8), KIF18A (HR = 1.7), KIF18B (HR = 1.7), KIF20A (HR = 2.2), KIF20B (HR = 1.8), and KIF23 (HR = 1.9) (**Figure 2**). On the contrary, the high expression of KIF21B (HR = 0.63) is associated with a better overall survival.

We also conducted a similar analysis using the LOGpc tool, an online web tool integrating multiple GEO and TCGA datasets, to reaffirm the association between high expression of KIFs genes and overall patient survival. A similar association between high expression of KIF23, KIF2C, KIF18B, KIF20A, KIF4A, KIFC1, KIF18A, KIF14, KIF11, KIF15, and KIF20B and shorter patient survival was conformed (**Supplementary Figure 2**). However, the association between KIF13B and KIF21B and patient survival does not seem significant. Next, we selected genes upregulated in PDAC and their association with prognosis in patients. KIFC1/2C/4A/11/14/15/18A/18B/KIF20A/20B/23 expression pattern was included in our further study. The KIF21B is a protective factor detected when using GEPIA tool, so it will be also included in our further analysis. We have named the 12-genes expression pattern as prognosis-related KIFs.

### Correlation Between Prognosis-Related KIFs and Tumor Grade in PDAC

**Figure 3:**
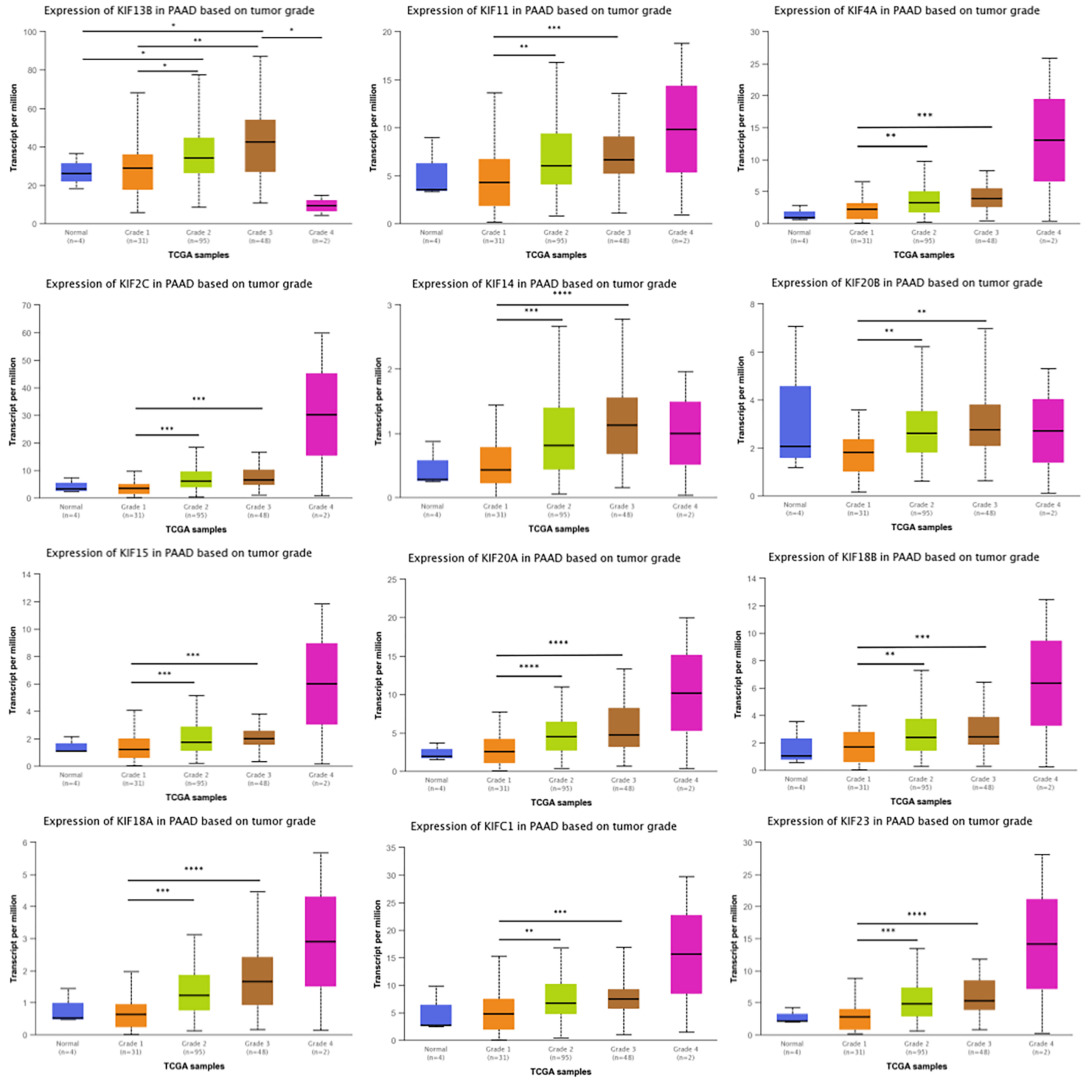
Correlation between prognosis associated KIFs expression and tumor grade in PDAC patients (UALCAN). With the increase in the KIFs mRNA expression, their tumor grade tended to be higher. The correlation between KIF2C, KIF4A, KIF11, KIF14, KIF15, KIF18A, KIF18B, KIF20A, KIF20B, KIF21B, KIF23, and KIFC1 and tumor grade in PDAC patients. *p < 0.05; **p < 0.01; ***p < 0.001; ****p < 0.0001.

Given that kinesin superfamily may be involved in the development of PDAC, we then assessed the correlation of kinesin superfamily expression and tumor grade in PDAC. We found that KIFs are significantly correlated with different grades of tumor, including KIFC1/2C/4A/11/14/15/18A/18B/20A/20B/23. The expression of KIFC1/2C/4A/11/14/15/18A/18B/20A/20B/23 increased in the comparison between grade 1 and grade 2/3 (**Figure 3**). We observed that the expression of KIF21B had decreased slightly between grade 1 and grade 2/3, although not statistically significant. In brief, we concluded that some of the kinesin superfamily members are closely tied to different grades of tumor and may play potential roles in tumorigenesis and development of PDAC.

### Kinesin Superfamily Genomic Alterations in Pancreatic Adenocarcinoma

**Figure 4:**
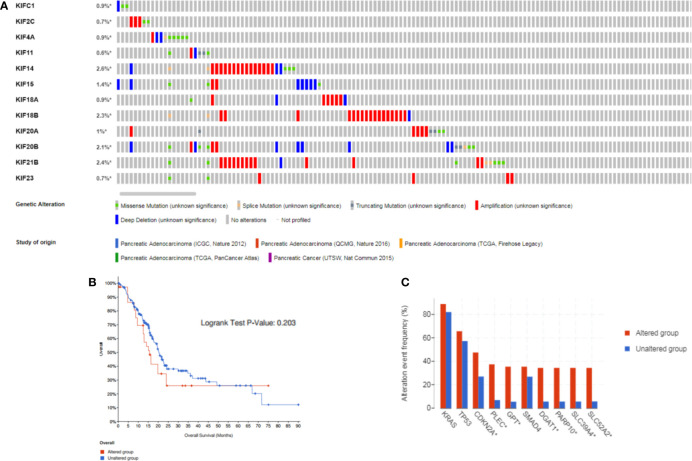
The gene alterations and correlations between prognosis-related KIFs. **(A)** cBioPortal OncoPrint of prognosis-related KIFs in PAAD cohort of the TCGA dataset. **(B)** Overall survival of PAAD patients with at least one alteration event of any prognosis-related genes and non-altered patients. **(C)** Comparison of most frequent gene mutation in PAAD patients with at least one alteration of any KIFs gene compared to non-altered patients.

Since the expression levels is the premise of function, we aimed to explore the kinesin superfamily genes highly expressed in pancreatic adenocarcinoma and their association with overall patient survival. The cBioPortal tool was employed to investigate genomic alterations of prognosis-related KIFs in TCGA pancreatic adenocarcinoma samples. The mutation landscape of prognosis-related KIFs is depicted in **Figure 4**. Ten percent of PDAC samples (84/850) harbored at least one kinesin superfamily gene alteration event, with amplification, mutation, and deep deletion being the most frequent alteration event (5.28%, 3.48%, and 1.93%, respectively). No significant statistical difference in survival was found between the altered and unaltered groups (*p* = 0.421; **Figure 4**). We also explored the different survival rates for individual kinesin superfamily genes in which KIF14/21B alterations were associated with poorer overall survival (**Supplementary Figures 3A, B** **)**. We then performed co-occurrence and mutually exclusive analysis for prognosis-related KIFs mutations. Most of the genes were co-occurrent with each other, in which KIF20B was co-occurrent with KIF2C, KIF11, KIF14, KIF15, KIF18B, and KIF20A. However, no statistical significance was observed when we performed mutually exclusive analysis among prognosis-related KIFs (**Supplementary Table 2**). Interestingly, we observed an enrichment of the cyclin-dependent kinase inhibitor 2A (CDKN2A) mutation in the prognosis-related KIFs gene-altered group (45.24% *vs.* 22.25%, p = 1.086e−5). Furthermore, gap junction protein (GJC2) (29.76% *vs.* 0.43%, p < 10^−10^), wingless-type MMTV integration site family, member 3A (WNT3A) (29.76% *vs.* 0.72%, p < 10^−10^), and Iron–Sulfur Cluster Assembly Factor IBA57 (IBA57) (27.38% *vs.* 0.43%, p < 10^−10^) were the most enriched gene mutations occurring in the KIFs altered group (**Figure 4C**).

### Prognosis-Related KIFs and KRAS and TP53 Mutations

**Figure 5:**
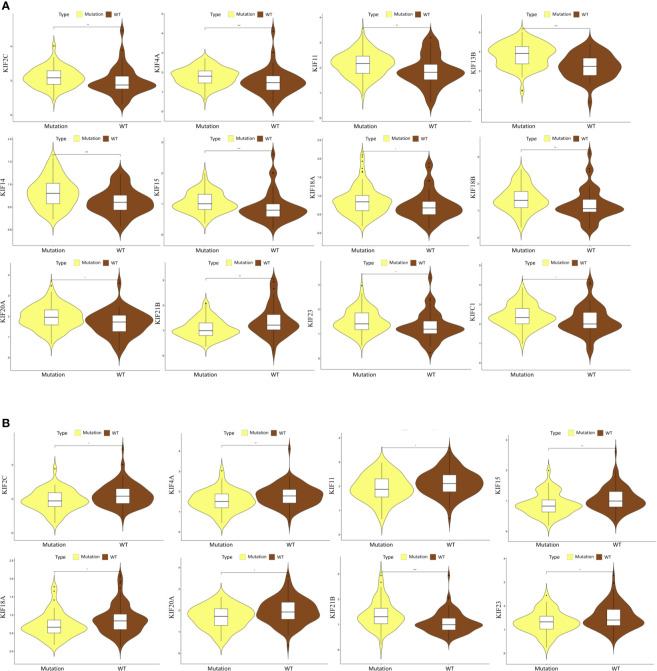
Correlation between prognosis-related KIFs expression and the mutation status of KRAS and TP53. **(A)** The correlation between KRAS mutation and the expression of prognosis-related KIFs. **(B)** The correlation between TP53 mutation and the expression of prognosis-related KIFs.

The mutations of two major driver genes, KRAS and TP53, are associated with malignant characteristics in pancreatic cancer. We then sought to clarify the relationship between the expression pattern of the kinesin superfamily and KRAS and TP53 mutations. We generated violin plots to visualize results. We identified that the expression of most kinesin superfamily members have significantly increased in those who have KRAS mutations (KIF23, KIF2C, KIF18B, KIF20A, KIF4A, KIFC1, KIF18A, KIF14, KIF11, KIF15, and KIF20B) (**Figure 5A**). On the contrary, KIF21B has significantly decreased in patients who have KRAS mutations. Only KIF20B did not harbor any significant difference between KRAS mutation and wild-type groups. A similar analysis was conducted to illustrate the relationship between the kinesin superfamily expression pattern and TP53 mutation. We noted that the expression of KIF23, KIF2C, KIF20A, KIF4A, KIF18A, KIF11, and KIF15 were lower in the TP53 mutation group (**Figure 5B**). However, no significant expression pattern was detected between KIF14, KIF18B, KIF20B, KIFC1, and TP53 mutation. Interestingly, we noticed an increased expression between KIF21B and TP53 mutation, indicating that it may be a protective indicator in pancreatic cancer.

### Correlation Between Prognosis-Related KIFs and Tumor Proliferation

**Figure 6:**
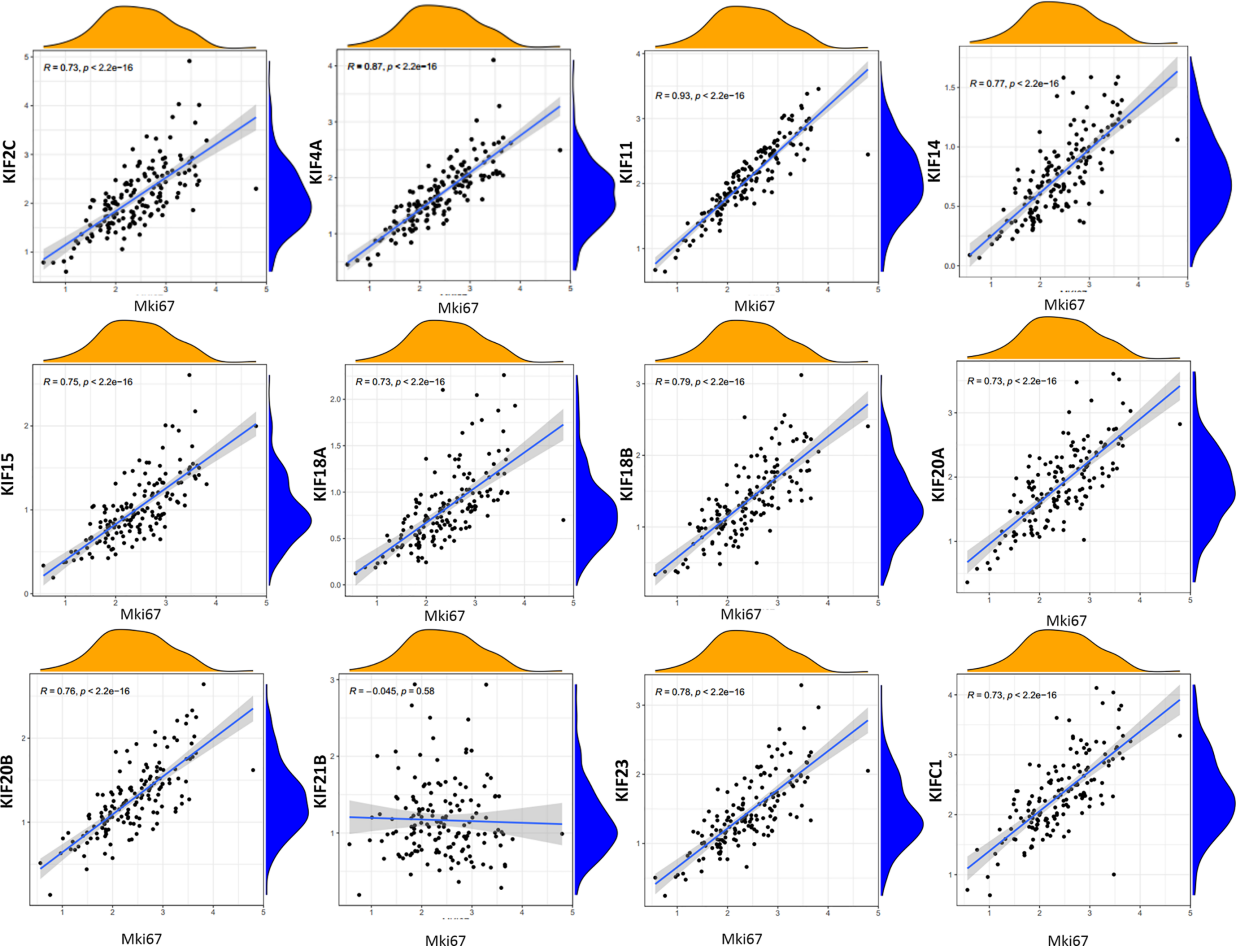
Correlation between prognosis-related KIFs expression and tumor growth. Scatter plot was presented to display the correlation between prognosis-related KIFs expression and Mki67. Pearson test was employed to evaluate the correlation coefficients. Pearson r > 0 mean positive correlation, Pearson r <0 means negative correlation. Pearson r = 0 means no correlation between two genes. *p* < 0.05 is considered to be statistically different.

To characterize the functional roles of the kinesin superfamily in cell proliferation, we calculated the correlation between prognosis-related KIFs and the well-known proliferation marker ki67 in PDAC. We identified a total of 11 significant associations, all of which were positive, suggesting the tumor-prompting role of prognosis-related KIFs. Multiple prognosis-related kinesin superfamily genes showed a strong correlation with Mki67, with KIF11, KIF4A, and KIF18B ranking the highest correlation coefficients (**Figure 6**). To further confirm the functional roles of prognosis-related kinesin superfamily genes in cell proliferation, we performed expression profiling analysis for 41 pancreatic cancer cell lines from CCLE and observed similar expression patterns (**Table 2**). To our surprise, KIF21B was negatively correlated with cell proliferation in the 41 pancreatic cancer cell lines, with the *p*-value of KIF21B being 0.0702. To conclude, we found that prognosis-related KIFs may play an indispensable role in cell proliferation.

**Table 2:** The correlation prognosis-related KIFs expression and tumor growth in 41 pancreatic cancer cell lines.

Symbol	Correlation	*p*-value
MKI67	1	0
KIF2C	0.524241964	4.35E−04
KIF4A	0.694311938	4.78E−07
KIF11	0.757149947	1.01E−08
		
KIF14	0.478460256	0.001554857
KIF15	0.650657405	4.12E−06
KIF18A	0.307471438	0.05051758
KIF18B	0.514215898	5.83E−04
KIF20A	0.735082912	4.43E−08
KIF20B	0.630896294	9.79E−06
KIFC1	0.579929763	7.07E−05
KIF23	0.638750771	6.99E−06
KIF21B	−0.28567361	0.070206767

### Correlation Between Prognosis-Related KIFs and Immune Infiltration

**Figure 7:**
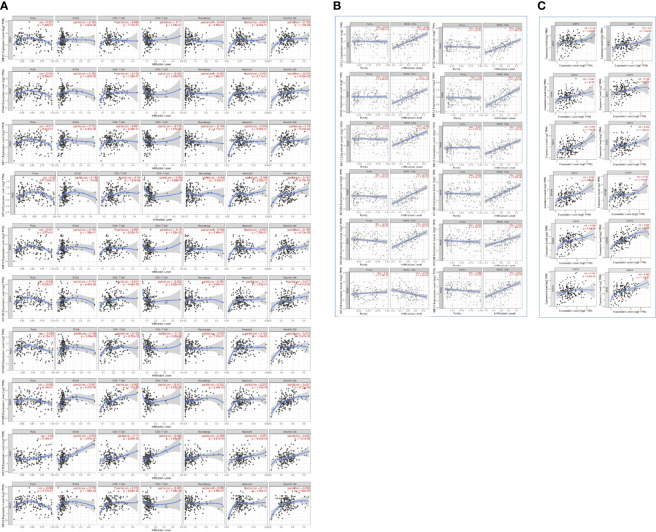
Correlation between prognosis-related KIFs expression and immune infiltration in PDAC patients (TIMER). **(A)** The correlation of 6 immunocytes with prognosis-related KIFs was estimated. **(B)** The correlation between MDSCs infiltration and prognosis-related KIFs. **(C)** The correlation between CD274 and prognosis-related KIFs. Cor > 0 represents a positive correlation, Cor < 0 represents a negative correlation. *P*< 0.05 means a significant correlation.

**Figure 8:**
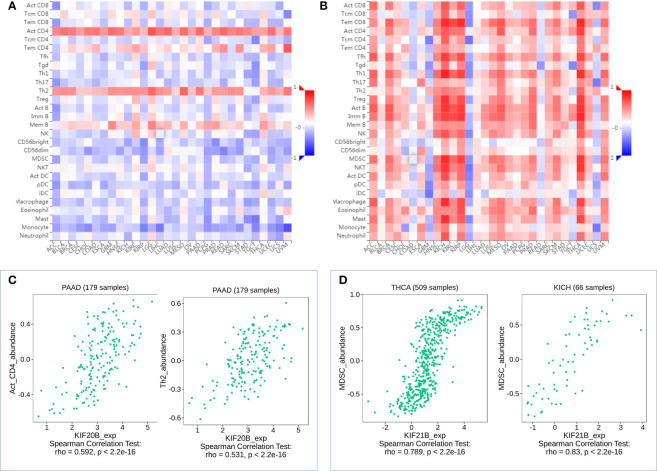
Correlation analysis of KIF20B, KIF21B level and immune cells infiltration levels across human cancers using the TISIDB database. **(A)** Relations between abundance of tumor-infiltrating lymphocytes (TILs) and expression of KIF20B. **(B)** Relations between abundance of tumor-infiltrating lymphocytes (TILs) and expression of KIF21B **(C)** KIF20B significantly correlated with abundance of Act_CD4 and Th2 in PDAC. **(D)** KIF21B significantly correlated with abundance of MDSC in TICH and KICH.

To further explore the roles of prognosis-related KIFs in cell–environment interaction, we performed a correlation of prognosis-related KIFs expression with immune infiltration level in PDAC. We found that 8 in 12 genes showed a positive correlation with B cell infiltration. Most of the prognosis-related KIFs had a positive correlation with dendritic cells infiltration. KIF14/18A/20B/21B/23 were positively correlated with CD8+ T cells infiltration, in which KIF20B have the highest Spearman’s rho value (**Figure 7A**). Interestingly, most of the prognosis-related KIFs had a negative correlation with CD4+ T cells infiltration, while KIF21B showed a positive correlation. However, KIF18B and KIFC1 showed no statistical significance in all kinds of immune cells. We then used TISIDB dataset to further explore the relationship between prognosis-related KIFs and 28 tumor immune-infiltrating cell subtypes. Interestingly, most of the prognosis-related KIFs expression were positively correlated with activated CD4 T cell and type 2 T helper cell (Th2) while negatively correlated with other immune infiltrating cell subtypes (**Supplementary Figure 4**). KIF21B showed a completely diverse immune landscape that was positively correlated with almost all kinds of immune infiltrating cell subtypes (**Figure 8**).

Myeloid-derived suppressor cells (MDSCs) are one of the most critical intratumoral suppressive myeloid cells in the stroma of PDAC. Thus, we investigated the relationship of prognosis-related kinesin superfamily genes and MDSCs’ level. To our surprise, most of those genes had remarkably positive correlations with MDSCs (**Figure 7B**). Only KIF21B exhibits a negative correlation with MDSCs infiltration, consistent with their role in prognosis. These results provided us new insights into the correlation between kinesin superfamily and immune infiltration in PDAC patients. The association between 12 prognosis-related kinesin superfamily genes and ICB therapy-related genes CD274 [also known as programmed death-ligand 1 (PDL1)] was also performed to assess the possible roles of those genes in the immunotherapy of ICB in PDAC. KIF23/2C/20A/4A/18A/14/11/15/20B were positively related to CD274, while KIF18B/C1/13B have no apparent relevance. Our findings may uncover a possible role of kinesin superfamily genes in ICB therapy (**Figure 7C**).

### The Construction of a Two-Gene Prognostic Model

**Figure 9:**
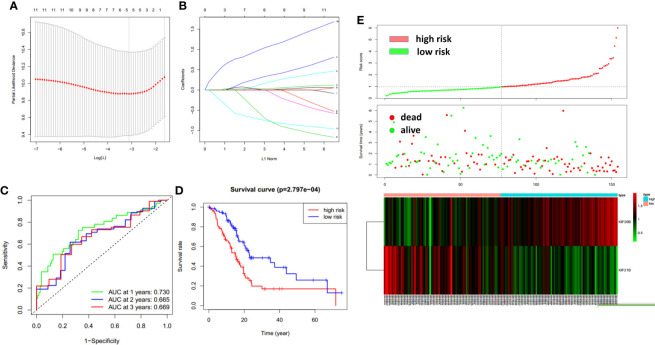
The construction of the two-gene prognostic model. **(A)** The changing trajectory of every single gene. The vertical axis represents coefficients of every single gene, and the horizon axis represents log (lambda) **(B)** The confidence interval at different value of λ. **(C)** Prognostic value of two-gene signature evaluated by ROC curves in TCGA-PAAD cohort. **(D)** Survival analysis of two-gene signature using Kaplan–Meier survival curves. **(E)** The distribution of survival status and expression profile of KIF20B and KIF21B between risk group.

To improve the prognostic ability of prognosis-related KIFs genes, a signature was constructed based on TCGA-PAAD cohort. One hundred fifty-four pancreatic cancer patients with prognosis information were enrolled in this study. According to univariate cox regression analysis, 11 genes (KIFC1 KIF2C KIF4A KIF11 KIF14 KIF18A KIF18B KIF20A KIF20B KIF21B KIF23) were correlated with patient overall survival. The hazard ratio and *p*-value are shown in **Table 3**. Next, least absolute shrinkage and selection operator (LASSO) regression was carried out using 10-fold cross-validation on 11 selected genes (**Figures 9A, B**). Then, five genes (KIF20A, KIF4A, KIF14, 20B, and KIF21B) produced by LASSO regression were then subjected to multivariate cox regression analysis. Ultimately, KIF20B and KIF21B were used to build a two-gene panel for predicting the survival of pancreatic cancer patients. Risk score was calculated as follows: (1.067*KIF20B exp.) + (−0.765*KIF21B exp.) According to the median of the risk score, we divided patients into a high- and a low-risk group. The distribution of survival status and expression profile of KIF20B and KIF21B between subgroups is presented in **Figure 9E**. Kaplan–Meier survival analysis revealed that patients with high scores have a dismal overall survival, indicating that the two-gene scores may be important indicators for pancreatic patients’ survival (**Figure 9D**). What is more, receiver operating characteristic (ROC) curves were utilized to evaluate the predictive power, and area under the curves (AUCs) for 1-, 2-, and 3-year OS were 0.730, 0.665, and 0.669, respectively **(** **Figure 9C** **).**

**Table 3:** Univariate and multivariate analyses of prognosis-related KIFs.

Characteristics	Univariate analysis			Multivariate analysis		
	HR	CI95	*p*-value	HR	CI95	*p*-value
KIF11	2.11	1.33–3.34	0.002	–	–	–
				–	–	–
KIF14	3.16	1.63–6.13	0.001	–	–	–
KIF15	1.61	0.98–2.65	0.062	–	–	–
KIF18A	2.11	1.29–3.46	0.003	–	–	–
KIF18B	1.53	1.04–2.25	0.032	–	–	–
KIF20A	2.07	1.38–3.1	0	–	–	–
KIF20B	3.22	1.8–5.75	0	2.42	1.18–4.96	0.016
KIF21B	0.41	0.24–0.71	0.002	0.44	0.25–0.76	0.004
KIF23	2.11	1.4–3.18	0	1.32	0.75–2.3	0.331
KIF2C	1.41	1.04–1.9	0.026	–	–	–
KIF4A	1.75	1.22–2.51	0.003	–	–	–
KIFC1	1.49	1.07–2.06	0.017	–	–	–

### Identification of Two-Gene Model as an Independent Prognostic Factor

**Figure 10:**
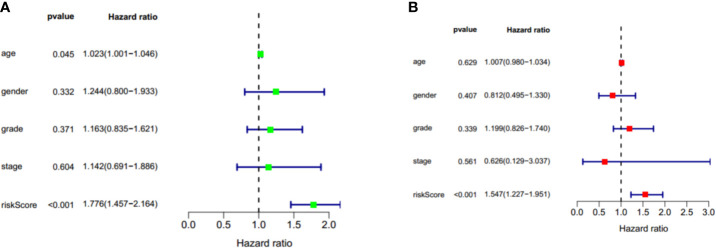
Univariate cox regression and multivariate cox regression analysis of two gene signature in TCGA-PAAD cohort. **(A)** Univariate cox regression analysis of two gene signature in TCGA-PAAD cohort. **(B)** Multivariate cox regression analysis of two gene signature in TCGA-PAAD cohort. Factors include age, gender, grade, stage, and riskscore. Hazard ratio>1 represent a risk factor while <1 represent a protective factor.

To identify whether the risk score could serve as the independent prognostic factor, TCGA-PAAD cohort with complete clinical information was used to perform univariate Cox regression analysis and multivariate Cox regression analysis. The results demonstrated that the two-gene signature was an independent prognostic factor (*p* < 0.001). The hazard ratios for OS were 1.776 and 1.547, respectively (**Figure 10**).

### The Protein–Protein Interaction of KIF20B, KIF21B, and Their Similar Proteins in PDAC

**Figure 11:**
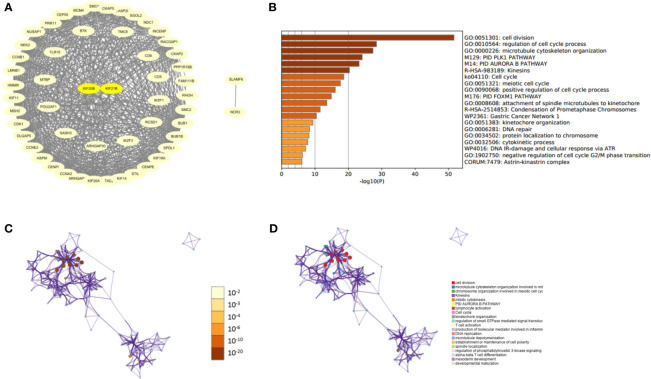
Protein-protein interaction network and functional enrichment analysis of KIFs in patients with PAAD (STRING and Metascape). **(A)** PPI of prognosis-related KIFs and their similar genes. **(B)** Prognosis-related KIFs genes ontology (G.O.) enriched terms, colored by *P*-values. **(C)** Network of G.O. enriched terms colored by *P*-value, in which terms containing more genes tend to have a more significant *P*-value. **(D)** Network of G.O. enriched terms colored by cluster-ID, where nodes share the same cluster-ID are typically close to each other.

After knowing the possible roles of KIF20B, KIF21B genes, we began to explore their similar genes in PDAC, which may play synergistic roles in PDAC development. The results of PPI networks demonstrated that those similar genes were closely tied to each other, which affirmed a synergistic function of comparable genes (**Figure 11A**). Gene Ontology (GO) analysis showed that those similar genes were significantly associated with cell division, cell cycle regulation, microtubule cytoskeleton organization, etc. (**Figures 11B–D**). Our results revealed that KIF20B and KIF21B and their similar genes play an essential role in cellular biological function.

### Gene Set Enrichment Analysis of KIF21B and KIF20B in PDAC

**Figure 12:**
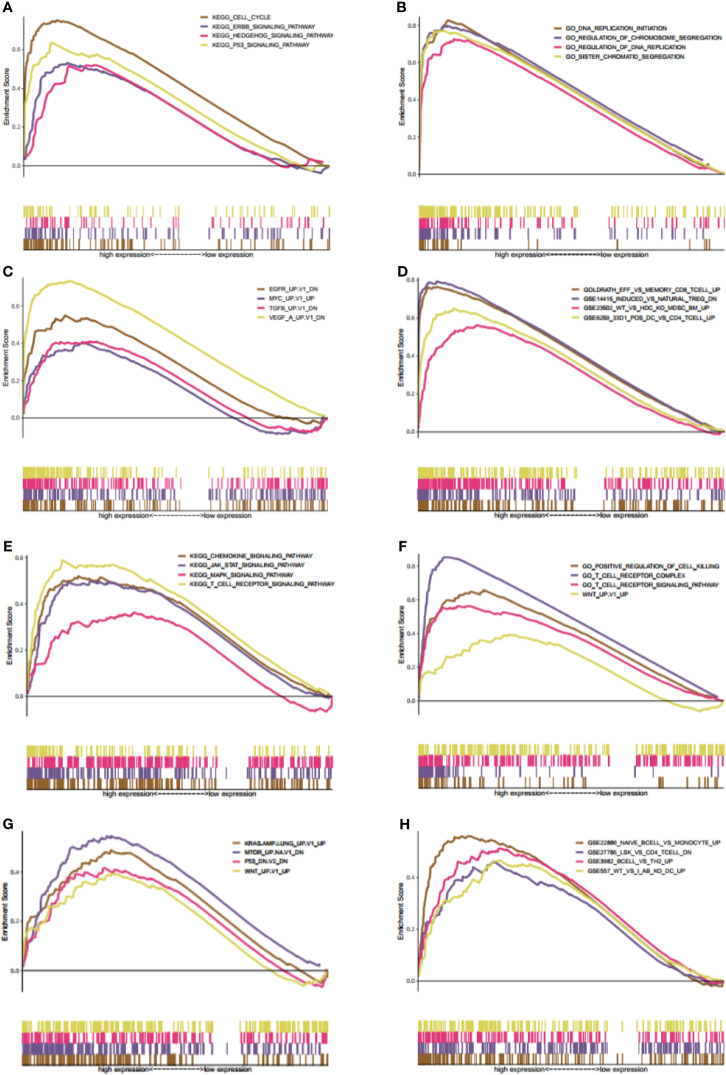
Gene set enrichment analysis (GSEA) of both KIF20B and KIF21B. **(A–D)** GSEA results of KIF20B expression in PC patients (**A–D** presents C2, C5, C6, C7 MSigDB respectively). **(E–H)** denote the GSEA of KIF21B **(E–H)** presents C2, C5, C6, C7 MSigDB respectively). The MsigDB C2, C5, C6, C7 were used to explore the oncogenic and immunologic roles of KIF20B and KIF21B. The GSEA results indicated that KIF20B and KIF21B were significantly enriched in oncogenic and immunologic pathway.

Gene set enrichment analysis was carried out to explore the possible mechanisms of different KIF20B and KIF21B expression levels affected clinical prognosis in PDAC patients. The MSigDB C2 (the curated gene sets), C5 (G.O. gene sets), C6 (oncogenic gene sets), and C7 (immunologic signature) were analyzed in our study. Enrichment of C2 demonstrated that high expression of KIF20B was involved in P53 signaling pathway, Hedgehog signaling pathway, and ERBB signaling pathway (**Figure 12A**). Enrichment of C5 showed that high expression of KIF20B was involved in DNA replication initiation, regulation of chromosome segregation, regulation of DNA replication, sister chromatid segregation (**Figure 12B**). Enrichment of C6 showed that high expression of KIF20B was involved in various oncogene signatures such as EGFR, VEGF, and MYC (**Figure 12C**). Enrichment of C2 demonstrated that increased expression of KIF21B was involved in the JAK-STAT signaling pathway, chemokine signaling pathway, T receptor signaling pathway, and MAPK signaling pathway (**Figure 12E**). Enrichment of C5 showed that high expression of KIF21B was involved in positive regulation of cell killing, T cell receptor complex, T cell receptor signaling pathway, WNT (**Figure 12F**). Enrichment of C6 showed that high expression of KIF21B involved in various oncogene signatures such as P53, KRAS, MTOM, and WNT (**Figure 12G**). Enrichment of C7 demonstrated that high expression of KIF20B and KIF21B was both involved in the immunologic process (**Figures 12D, H**). Accidentally, we found that KIF2C/4A/11/14/15/16B/20A/22/23/25 were core enriched in immunologic signature (**Supplementary Table 1**).

### The Expression Patterns of KIF20B and KIF21B in Pancreatic Cancer Cell Lines

**Figure 13:**
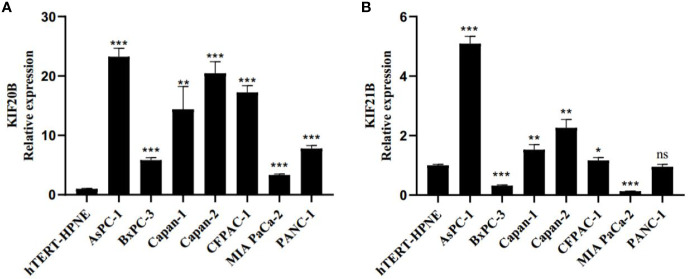
Real-time quantitative PCR (RT-qPCR) evaluate the expression pattern of KIF20B and KIF21B in pancreatic cancer cell lines and normal pancreatic cell. **(A)** The expression pattern of KIF20B in pancreatic cancer cell lines and normal pancreatic cell. **(B)** The expression pattern of KIF21B in pancreatic cancer cell lines and normal pancreatic cell. ns, not significant. “*” denote p < 0.05; “**” denote p < 0.01; “***” denote p < 0.001; “****” denote p < 0.0001.

Next, we investigated the gene expression of KIF20B and KIF21B in pancreatic cancer cell lines and normal pancreatic cell. We found relatively higher expressions of KIF20B in ASPC-1, BxPC-3, Capan-1, Capan-2, CFPAC-1, MIA PACA-2, and PANC-1 compared that in hTERT-HPNE **(** **Figure 13A** **)**. The expression of KIF21B was relatively higher in ASPC-1 Capan-1, Capan-2, CFPAC-1 compared to that in hTERT-HPNE **(** **Figure 13B** **)**. However, the expression level of KIF21B in BxPC-3 and MIA PACA-2 presented a relatively lower expression. In conclusion, KIF20B and KIF21B were highly expression in most pancreatic cancer cell lines compared to normal pancreatic cell.

## Discussion

Including PDAC, numerous studies have indicated that KIFs participate in biological functions such as intracellular transportation, cell division, occurrence, and development of many tumors (4, 5). For example, KIF23 promotes cell proliferation in PDAC and is a potent therapeutic target (18). KIF18B promotes the proliferation of PDAC by activating the expression of CDCA8. Although some KIFs genes have been investigated in PDAC (43), there were no reports of different KIFs expression and their role in PDAC. As far as we know, we are the first to systematically investigated prognostic values, genetic variation, and tumor-promoting potential of different KIFs in PDAC. Moreover, for the first time, we demonstrate that the overexpression of KIF20B and KIF21B may promote immune suppression of PDAC by activating MDSCs. More importantly, we revealed the possible molecular mechanism involving the immunosuppression of PDAC and may provide new targets for immune therapy. Although previous study has investigated the prognostic value of Kinesin-4 family genes, our study uncovered the immune infiltration roles of prognosis-related KIFs and comprehensively investigated the tumor-promoting potential of different prognosis-related KIFs in PDAC.

Tumor grade is one of the most important parameters used to judge the degree of malignancy. In this investigation, we evaluated the prognosis values of the 12 prognosis-associated KIFs. Interestingly, most of those genes were strikingly lower in grade 1 than those in grade 2/3 but not in grade 4. It indicated that the expression levels of KIFs could be good indicators of patient prognosis. Interestingly, the association between higher expression of KIF21B and a better clinical outcome indicated that KIF21B might be a good indicator in PDAC prognosis. The KIFs have been widely reported to play fundamental roles in intracellular transportation, such as in organelle, macromolecule, and messenger RNA (mRNA) (5). When tumor displays increased invasive and metastatic potential, the metabolic demand gets improved accordingly by upregulation of metabolic-related genes, such as KIFs. There is another explanation that insufficient samples of normal tissue and grade 4 result in their non-significance with other stages. Thus, further studies should be carried out to elucidate the potential roles of prognosis-associated KIFs in PDAC.

Based on the correlation between Ki67 and prognosis-associated KIFs, our study indicated that most of the genes are highly implicated in tumor growth, which is consistent with previous findings. Gu et al. suggested that knockdown of KIF26B suppressed breast cancer cell growth and invasion (17). KIFC1 was implicated in the progression of NSCLC by regulating the cell proliferation and cell cycle (44). KIF20B was not only related to tumor sizes and T stage but also promoted the progression of ccRCC by stimulating cell proliferation (19). GSEA results demonstrated that high expression of KIF20B was significantly associated with cell division, ERBB signaling pathway, and Hedgehog signaling pathway, which is in line with their role in regulating the cell proliferation. However, up to now, most of KIFs have not been systematically assessed regarding their potential abilities in supporting PDAC cell proliferation. Thus, our study provided deeper insight into the potential mechanisms of KIFs in pancreatic carcinogenesis.

The mutations of two major driver genes, KRAS and TP53, were associated with malignant characteristics in pancreatic cancer. The KRAS mutations lead to constant activation of KRAS and persistent stimulation of its downstream signaling pathways that drive many of the hallmarks of cancer, sustained proliferation, metabolic reprogramming, antiapoptosis, remodeling of the tumor microenvironment, evasion of the immune response, cell migration, and metastasis (45). Our study found that KIFs are significantly upregulated in patients with KRAS mutations, indicating that there are some complex interactions between KRAS signaling pathway and KIFs. Our GSEA results also showed that KIF20B was implicated in the MAPK signaling pathway, an essential downstream KRAS signaling pathway. On the contrary, most genes are significantly downregulated in patients with TP53 mutation.

Interestingly, KIF21B was upregulated in patients with TP53 mutations while downregulated in patients with KRAS mutations. The results from GSEA also showed that high expression is strikingly enriched in the TP53 signaling pathway. An experimental study will be performed to explore the role of KIFs in the KRAS signaling pathway and TP53 signaling pathway during pancreatic carcinogenesis in our future work.

Despite rampaging evidence has shown the clinical benefits of immune-targeted approaches, checkpoint-based immunotherapy has failed to elicit responses in the vast majority of patients with pancreatic cancer (3). The immunological role of prognosis-related KIFs was investigated in some studies. For examples, KIF20A has shown its roles in PDAC immune therapy (46). However, studies on the immune suppression of KIFs in pancreatic cancer have not been performed to date. In this study, we explored the immune infiltration role of the 12 prognosis-related KIFs. The results indicated that 8/13 genes were positively correlated with B-cell infiltration. Increasing evidence revealed that B-cell subsets in PDAC tumor microenvironment upregulate associated immunosuppressive cytokines (most notably, IL-10, IL-18, and IL-35) and immune checkpoint ligands (particularly PD-L1), contributing to oncogenesis and the inhibition of T cell-mediated tumor immunity (47). Interestingly, we also found a positive correlation between prognosis-related KIFs and PD-L1. We also investigated the immune infiltration roles of prognosis-related KIFs. The results proved that prognosis related KIFs were correlated with immune infiltration in pan-cancer.

Myeloid-derived suppressor cells (MDSCs) are among the most important immunosuppressive cells in stroma of PDAC, which directly induces the T-cell suppression by secreting factors and indirectly by inducing tumor-cell-specific PD-L1 expression to inhibit spontaneous antitumor immunity (48). In our study, the correlation between infiltration levels of MDSCs and prognosis-associated KIFs was also explored. Most of those genes were significantly correlated with the infiltration level of MDSCs. Numerous studies have examined the pathways that regulate MDSCs. Thomas Welte et al. indicated that mTOR signaling recruits myeloid-derived suppressor cells to promote tumor initiation (49). Nikolaos Svoronos et al. found that tumor-cell-independent estrogen signaling enhanced pSTAT3 activity through transcriptional upregulation of JAK2 and increased total STAT3 expression in myeloid progenitors and therefore drove disease progression through mobilization of myeloid-derived suppressor cells (50). GSEA also affirmed the correlation between KIF20B and MDSCs.

Moreover, GSEA showed that KIF20B was significantly enriched in the mTOR oncogenic and estrogen signaling pathway. Similar results showed that KIF21B was improved considerably in the chemokine signaling pathway, JAK-STAT signaling pathway, and T-cell signaling pathway. All these results indicated that the KIFs might regulate MDSCs in stroma by activating the mTOR oncogenic and estrogen signaling pathway and therefore mediate the immune suppression of PDAC. Consequently, we propose that KIF20B and KIF21B play an indispensable role in the immune suppression of PDAC, which could be promising therapeutic targets complementary to current immunotherapies.

In conclusion, we integrated analysis of the 12 prognosis-associated KIFs by utilizing multiple datasets and confirmed their involvements in the development and progression of PDAC. The expression pattern was first identified, followed by a series of correlation analysis. The results showed that 13 prognosis-associated KIFs were strongly correlated with tumor stage, immune infiltration, cell growth, and mutation status of KRAS and TP53. A two-gene prognostic model was used to predict the prognosis of PDAC based on each patient’s risk score. ROC curves evaluated the predictive power of two-gene signature. PPI of two genes and their similar genes and GSEA demonstrate that KIF20B and KIF21B were both implicated in immune regulation and oncogenic processes. KIF20B and KIF21B could be promising immunological therapeutic targets. However, there do exist some limitations in our study. The first limitation of this study is the insufficiency of validating external clinical cohort and experimental validation. Second, enough normal and stage 4 samples should be utilized to evaluate the prognostic value of KIFs.

## Data Availability Statement

The datasets presented in this study can be found in online repositories. The names of the repository/repositories and accession number(s) can be found in the article/**Supplementary Material**.

## Author Contributions

QZ, YY conceived the project and wrote the manuscript. LG, N-NW, J-JL, and YZ participated in data analysis. AV-R participated in discussion and language editing. RL, Q-LX, Y-FX, and JL reviewed the manuscript. All authors contributed to the article and approved the submitted version.

## Funding

Grants 5G12MD007592 and 2U54MD007592 sponsored AV-R from the Research Centers in Minority Institutions (RCMI) program to the Border Biomedical Research Center (BBRC) at the University of Texas at El Paso (UTEP), from the National Institute on Minority Health and Health Disparities (NIMHD), a component of the National Institutes of Health.

## Author Disclaimer

This manuscript’s contents are solely the authors’ responsibility and do not necessarily represent the official views of BBRC, UTEP, RCMI, NIMHD, or NIH.

## Conflict of Interest

The authors declare that the research was conducted in the absence of any commercial or financial relationships that could be construed as a potential conflict of interest.

## Publisher’s Note

All claims expressed in this article are solely those of the authors and do not necessarily represent those of their affiliated organizations, or those of the publisher, the editors and the reviewers. Any product that may be evaluated in this article, or claim that may be made by its manufacturer, is not guaranteed or endorsed by the publisher.
