# Supplementary material for: Identification of Novel Molecular Therapeutic Targets and Their Potential Prognostic Biomarkers Among Kinesin Superfamily of Proteins in Pancreatic Ductal Adenocarcinoma
Source: Front Oncol. 2021 Sep 7;11:708900. doi: 10.3389/fonc.2021.708900 (PMC8454465; doi:10.3389/fonc.2021.708900)
Supplement: Supplementary file 1 [file DataSheet_1.doc]

**Supplementary table 1** | KIFs are enriched in immunologic gene sets.

| Symbol | Title | Rank in gene list | Rank metric score | Running E.S. | Core enrichment | Gene sets |
| --- | --- | --- | --- | --- | --- | --- |
| KIF11 | n/a | 6 | 0.799594522 | 0.0336974 | Yes | Gene set 1 |
| KIF23 | n/a | 17 | 0.736834407 | 0.08017901 | Yes |
| KIF20A | n/a | 30 | 0.699155331 | 0.16748121 | Yes |
| KIF4A | n/a | 38 | 0.663122535 | 0.20885564 | Yes |
| KIF11 | n/a | 6 | 0.799594522 | 0.05585471 | Yes | Gene set 2 |
| KIF15 | n/a | 58 | 0.622734427 | 0.19713376 | Yes | Gene set 3 |
| KIF14 | n/a | 1 | 0.8790555 | 0.0178461 | Yes |
| KIF11 | n/a | 6 | 0.799594522 | 0.06851755 | Yes |
| KIF23 | n/a | 17 | 0.736834407 | 0.16060561 | Yes |
| KIF4A | n/a | 38 | 0.663122535 | 0.31805295 | Yes |
| KIF15 | n/a | 58 | 0.622734427 | 0.42132992 | Yes |
| KIF2C | n/a | 179 | 0.46639955 | 0.61709744 | Yes |
| KIF22 | n/a | 3293 | 0.203867942 | 0.7832755 | Yes |
| KIF16B | n/a | 11515 | 0.140401423 | 0.47612923 | Yes | Gene set 4 |
| KIF25 | n/a | 13510 | 0.125574559 | 0.4967958 | Yes |

n/a = not available

Gene set 1: “GSE6259_33D1_POS_DC_VS_CD4_TCELL_UP”

Gene set 2: “GSE6259_33D1_POS_DC_VS_CD4_TCELL_UP”

Gene set 3: “GSE14415_INDUCED_VS_NATURAL_TRE”

Gene set 4: “GSE3982_BCELL_VS_TH2_UP”

**Supplementary table 2** | Co-occurrence and mutually exclusive analysis for KIFs mutations.

| A | B | Neither | A  Not  B | B Not  A | Both | Log2 Odds  Ratio | p-Value | tendency |
| --- | --- | --- | --- | --- | --- | --- | --- | --- |
| KIF2C | KIF14 | 715 | 4 | 18 | 3 | >3 | <0.001 | Co-occurrence |
| KIF14 | KIF15 | 712 | 16 | 7 | 5 | >3 | <0.001 | Co-occurrence |
| KIFC1 | KIF15 | 723 | 5 | 10 | 2 | >3 | 0.005 | Co-occurrence |
| KIF2C | KIF15 | 723 | 5 | 10 | 2 | >3 | 0.005 | Co-occurrence |
| KIFC1 | KIF18B | 718 | 5 | 15 | 2 | >3 | 0.01 | Co-occurrence |
| KIF15 | KIF20B | 715 | 6 | 13 | 6 | >3 | <0.001 | Co-occurrence |
| KIF11 | KIF20B | 720 | 1 | 15 | 4 | >3 | <0.001 | Co-occurrence |
| KIF14 | KIF20B | 706 | 15 | 13 | 6 | >3 | <0.001 | Co-occurrence |
| KIF18A | KIF20B | 716 | 5 | 16 | 3 | >3 | <0.001 | Co-occurrence |
| KIF18B | KIF20B | 707 | 14 | 16 | 3 | >3 | 0.008 | Co-occurrence |
| KIF2C | KIF20B | 716 | 5 | 17 | 2 | >3 | 0.012 | Co-occurrence |
| KIF20A | KIF20B | 716 | 5 | 17 | 2 | >3 | 0.012 | Co-occurrence |
| KIF14 | KIF21B | 708 | 13 | 11 | 8 | >3 | <0.001 | Co-occurrence |
| KIF18B | KIF21B | 708 | 13 | 15 | 4 | >3 | <0.001 | Co-occurrence |
| KIF14 | KIF23 | 717 | 18 | 2 | 3 | >3 | <0.001 | Co-occurrence |
| KIF15 | KIF23 | 725 | 10 | 3 | 2 | >3 | 0.002 | Co-occurrence |
| KIFC1 | KIF2C | 728 | 5 | 5 | 2 | >3 | 0.002 | Co-occurrence |


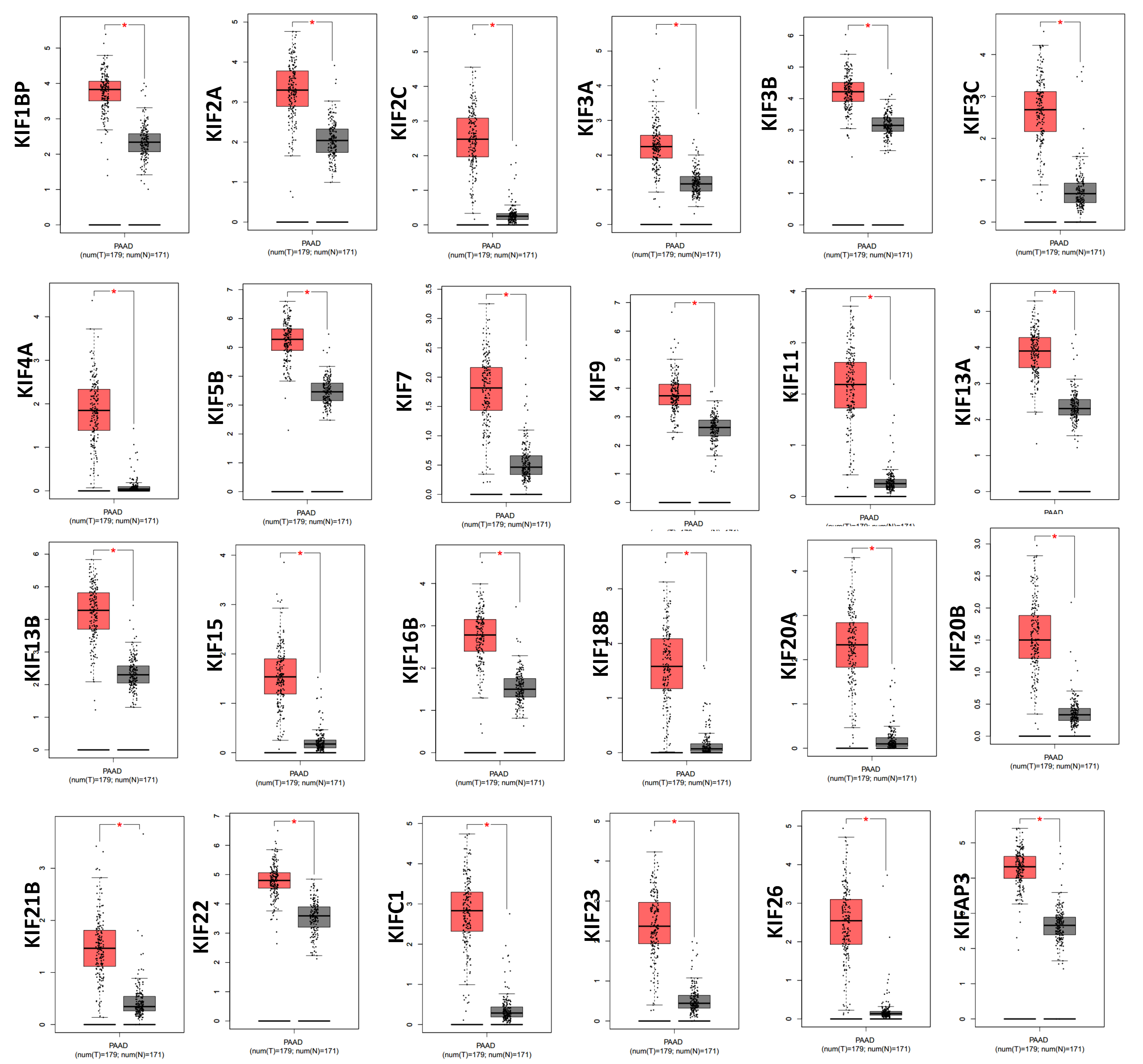


**Supplementary Figure 1** | The aberrant expression of KIFs in PDAC patients via the GEPIA. The significantly up-regulated KIFs were shown in box plots. Student t test was employed to found the aberrant expression of KIFs in PDAC patients. P<0.05 was considered statistically significant.


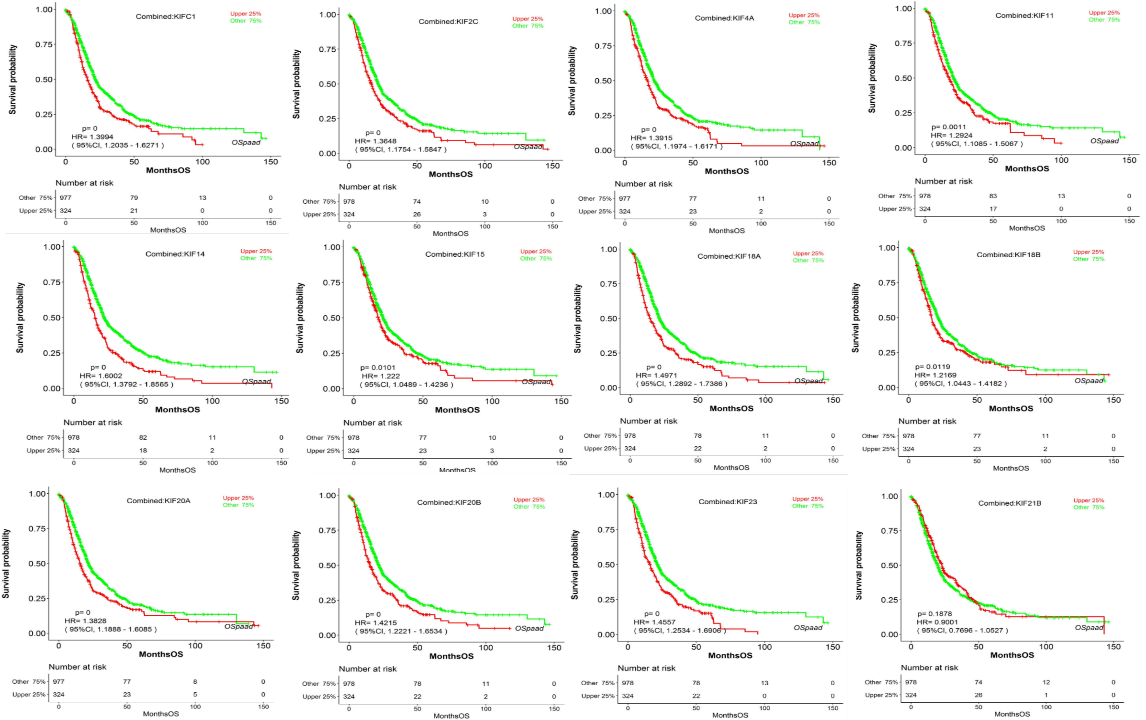


**Supplementary Figure 2** | The prognostic values of KIFs in PDAC patients in the OS curve (LOGpc). All the prognostic analysis was performed by integrating multiple datasets.

#
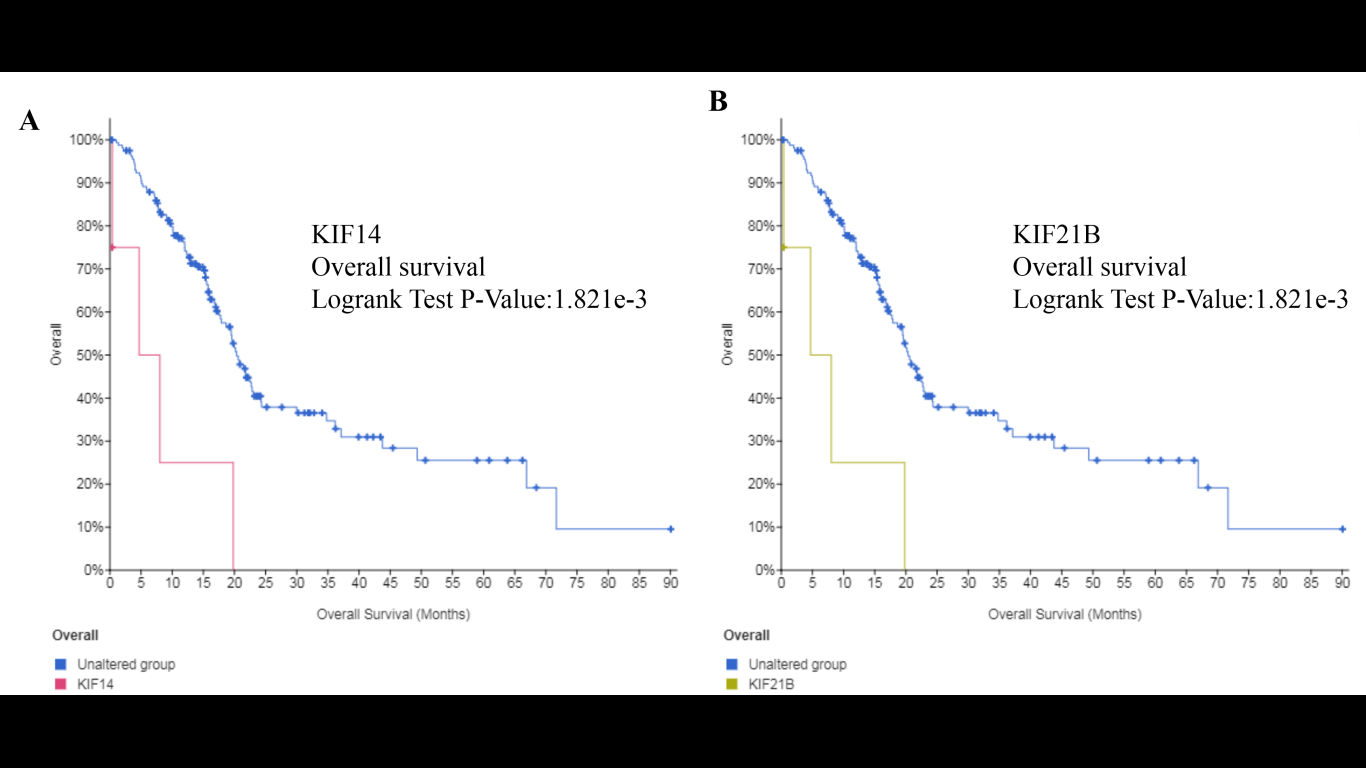


**Supplementary Figure 3** | Overall survival of PDAC patients between KIFs alteration and non-altered patients. **(A)** Overall survival of PDAC patients with at least one alteration event of KIF14 and non-altered patients. **(B)** Overall survival of PDAC patients with at least one alteration event of KIF21B and non-altered patients.


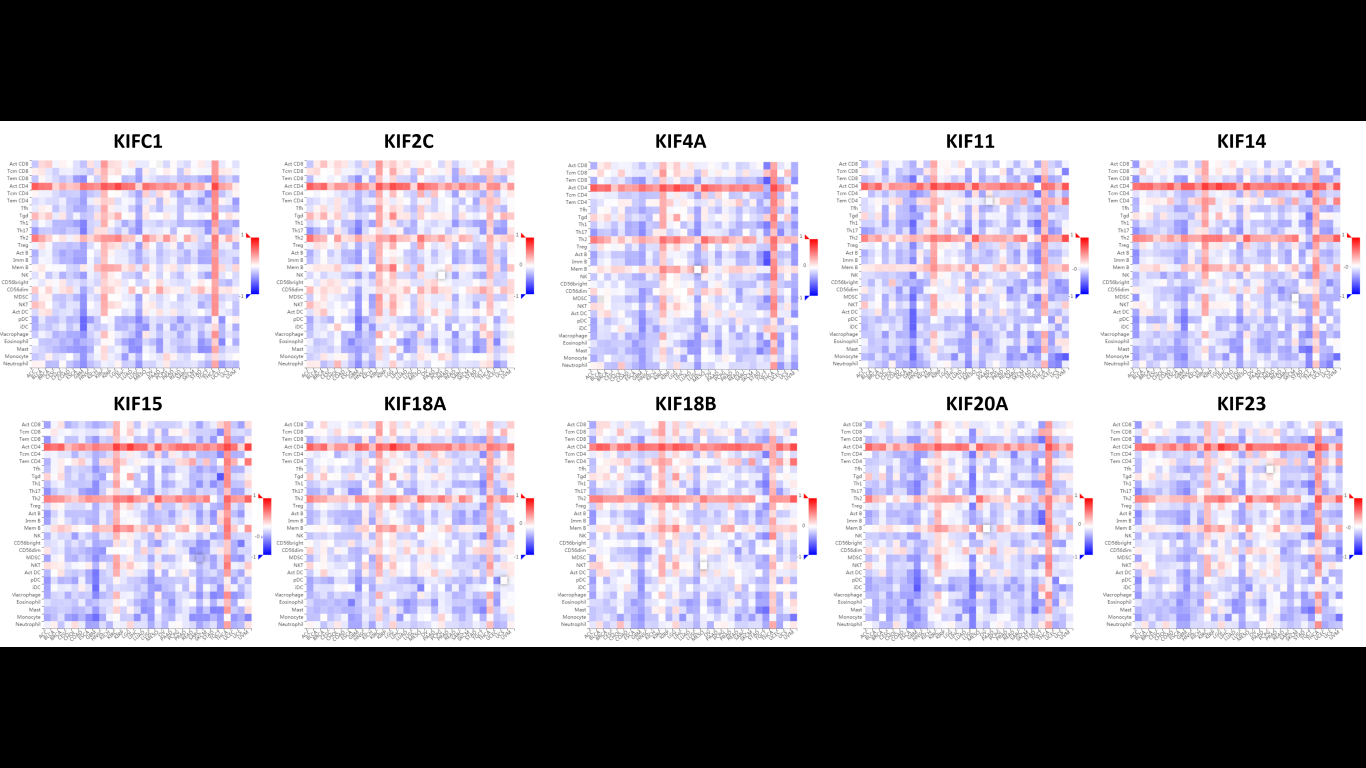


**Supplementary Figure 4** | Correlation analysis of prognosis-related KIFs level and immune cells infiltration levels across human cancers using the TISIDB database.

**
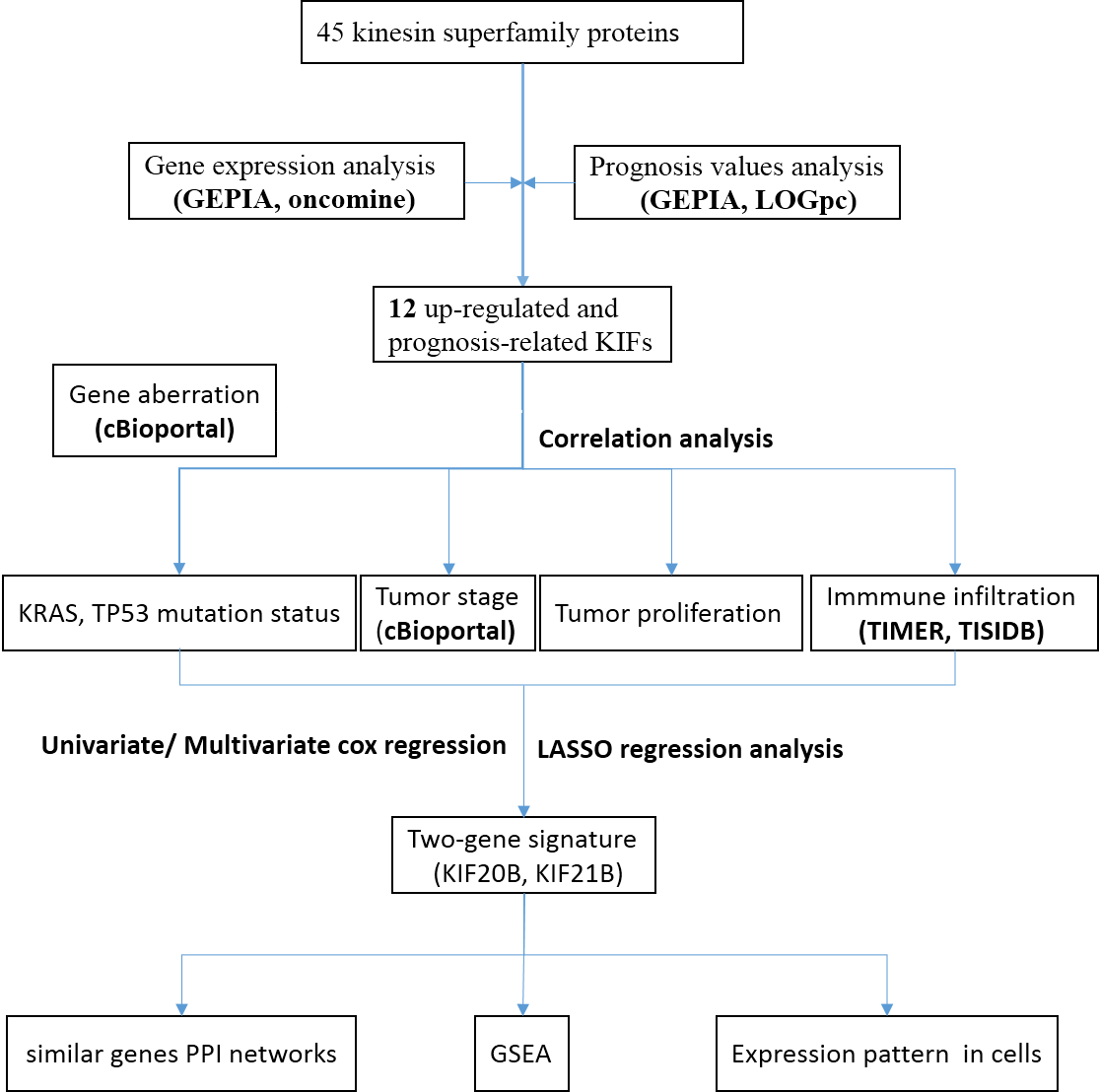
**

**Supplementary Figure 5** | A guided flowchart of the strategy and methodology utilized in this study.
